# Multi-level determinants of physical activity and sports participation among adults during COVID-19 pandemic: an interpretable machine learning approach

**DOI:** 10.3389/fpsyg.2025.1701201

**Published:** 2026-01-07

**Authors:** Kai Zhao, Zehong Chen, Qian Huang, Shuting Li, Guangxin Tan, Kai Guo, Lilei Jiang

**Affiliations:** 1 School of Economics and Management, Chengdu Sport University, Chengdu, China; 2 School of Physical Education and Health, Guangdong Polytechnic Normal University, Guangzhou, China; 3 School of Intelligent Sports Engineering, Wuhan Sports University, Wuhan, China; 4 School of Economics and Management, Wuhan Sports University, Wuhan, China; 5 School of Physical Education and Sports Science, South China Normal University, Guangzhou, China; 6 School of Management, Beijing Sport University, Beijing, China

**Keywords:** adults, COVID-19, influencing factors, interpretable machine learning, physical activity, socio-ecological model, sports participation

## Abstract

**Background:**

Both physical activity (PA) and sports participation (SP) are considered important for the promotion of health among adults in the post-disease outbreak period. In the context of the COVID-19 pandemic, the study applied the Socio-ecological Model, with a total of 45 factors on four levels: individual characteristics, individual behaviors, interpersonal relationships, and community environment. The aim was to apply interpretable machine learning algorithms in the examination of common and distinct determinants of PA and SP with the purpose of deriving specific insights relevant to public health policy.

**Methods:**

To examine the comparable but different patterns of behavior regarding PA and SP, this research used the Chinese General Social Survey of 2021 with a sample of N = 2,717 participants. Eight machine learning models were designed with the aid of Python coding, including the following models: Logistic Regression, Support Vector Machine, Decision Tree, Random Forest (RF), Adaptive Boosting, Gradient Boosting Decision Tree, eXtreme Gradient Boosting Model (XGBoost), and Light Gradient Boosting. As part of evaluating these models' performance, Accuracy, Area Under the Curve (AUC), and the F1-score results were used after executing the grid search on the models' respective variables. The Permutation Feature Importance method was used to quantify factor importance and identify key factors, and Partial Dependence Plots were generated to interpret the direction of these influences.

**Results:**

Results showed that the best algorithm for predicting PA was the RF with an AUC of 0.613 and that it selected 10 key factors. Additionally, the best algorithm that predicted SP was XGBoost with an AUC of 0.772, and it selected 12 key factors. Common influencing factors during the COVID-19 pandemic include suitability for exercise and recreational lifestyle, with BMI category also playing a significant role. Distinctive factors of PA were primarily related to the community environment (e.g., fresh food outlets and neighborhood care), reflecting its dependence on environmental contexts. In contrast, distinctive factors of SP were more concentrated at the individual characteristics (e.g., education level and socioeconomic status) and behaviors level (e.g., learning and health examination), highlighting the role of personal initiative and the accumulation of socio-cultural and economic capital.

**Conclusion:**

The Socio-ecological Model effectively delineated commonalities as well as differences in determinants of PA and SP across adults during the COVID-19 pandemic. Interpretable machine learning aided in identifying and ranking multi-level determinants, offering a nuanced insight into the relative importance across levels of ecology. These findings provide data-driven insights for future disease outbreaks, facilitating the targeted allocation of intervention resources to key influencing domains.

## 1 Introduction

Physical activity (PA) and sports participation (SP) are essential to ensuring adult health and well-being. There is a considerable body of evidence that indicates that PA on a regular basis benefits physical health (Warburton et al., 2006), and also has a positive impact on mental health and wellbeing (Mahindru et al., 2023). In addition, SP enables social capital development (Perks, 2007) and healthier lifestyles (Palomäki et al., 2018). In recognition of its widespread influence, sport was included in The Agenda 2030 for sustainable development as a strategic instrument to promote the Sustainable Development Goals (SDGs) (Dai and Menhas, 2020). PA and SP have also been given priority by China within its national development strategy (Li et al., 2023). However, against the backdrop of the COVID-19 pandemic, PA levels have decreased across all population groups, leading to declining health and fitness status (Eshelby et al., 2022). Physical inactivity and low participation in sports have proven prevalent and have a tendency to persist from adolescent through adult life (Hirvensalo and Lintunen, 2011). The Global Status Report on PA 2022 (World Health Organization, 2023) further states that nearly 27.5% of all the adults worldwide, covering a total of 1.4 billion people, are not attaining a sufficient level of PA. Without effective intervention, the global target of reducing physical inactivity by 15% by 2030 is unlikely to be achieved (Strain et al., 2024).

The challenges are further compounded by a health transition driven by rapid industrialization, urbanization, and increasingly sedentary lifestyles (Menhas et al., 2021). Accordingly, a systematic analysis of key factors influencing physical inactivity and sport participation in adults during the COVID-19 pandemic is therefore essential in addressing said challenges and preparing for future disease outbreaks. Existing literature found that participation in PA and sports during the COVID-19 pandemic is determined by a variety of factors on several levels, that is, by individual characteristics (e.g., sex, age) (Pelletier et al., 2021), by individual behaviors (e.g., health behaviors) (Lin and Liu, 2023), by interpersonal relationships (e.g., support from peers and family) (Van Luchene and Delens, 2021; Wang et al., 2022), as well as by community factors (e.g., environmental attributes) (Laddu et al., 2021; Lee et al., 2024). They are consistent with the multi-level structure of the Socio-ecological Model (McLeroy et al., 1988), which posits that health behaviors arise as a result of interactive processes between individuals and proximal environments (Song et al., 2021). More specifically, the Socio-ecological Model further breaks down the variables that affect health behavior into four major levels: the microsystem, mesosystem, exosystem, and macrosystem (Spence and Lee, 2003). The microsystem is considered the immediate system that surrounds the individual, the mesosystem is made up of the interactions of two or more microsystems, the exosystem is the bigger system that can affect the individual and their environment through several different methods and channels of transmission, and the macrosystem is the most remote system of the four levels and comprises the combined effects of the first three levels (Bronfenbrenner, 1977). By applying the theoretical part of the Socio-ecological Model to the system proposed above aimed at encouraging adult PA and SP during the COVID-19 pandemic crisis, the levels of the system could be defined as follows: the microsystem would encompass the individual's personal characteristics that affect and modify their health behavior, the individual's behavior patterns because of their interactions with the surrounding environment and the interactions of other different microsystems like the family environment and friends/peers, the organizational environment of the individual's workplace and the surrounding community, and the individual's and community's sociological environment that modifies their behavior patterns according to the policies regarding PA and SP involvement. Therefore, in the field of health behavior, scholars generally specify these systems into concrete environments such as individual, interpersonal, organizational, community, and policy levels (McLeroy et al., 1988). Hence, a multilevel theory is necessary in order to explain the antecedents of taking part in PA and sport during the COVID-19 pandemic. Nevertheless, there may still exist too much specialization on the former research on PA or SP individually (Crossman et al., 2024). Though PA and SP can both belong to health behavior (Hirvensalo and Lintunen, 2011), their definition are different (Beunen and Thomis, 1999). As the definition given by Caspersen et al. (Caspersen et al., 1985), the explanation of PA could be the voluntary movement produced by the contraction of the skeletal muscles that results in an increase of the expenditure of energy, while SP could be the subset of PA that must possess characteristics of its being planned, supervised, and repeated with the purpose of developing increased fitness levels regarding one or more elements of the physical fitness patterns. The evidence provided showed that there are considerable distinctions in the rate of PA and SP among the different countries (Lera-López and Marco, 2018), hence the relative research on them compares and analyzes the results of the PA and SP together, hopefully aiming at formulating more precise intervention programs (Tammelin et al., 2003; Brown, 2005). Moreover, the relative research may just concentrate on the unilevel elements (Lee and Park, 2021), or be based on the Western group only, with few undertaken on the Asian group (Huang et al., 2024). More serious could be the lack of the relative research on adults' PA behavior that is based on the socio-ecological pattern during the COVID-19 pandemic (Zhang et al., 2024a).

The fragmented understanding of factors influencing adults' PA and SP during the COVID-19 pandemic has, in part, been attributed to the methodological limitations of traditional statistical approaches (Fu et al., 2024). While traditional causal inference methods have contributed to the development of a mature research paradigm, they often require a strong theoretical foundation (Lemon et al., 2003). They also face several constraints, such as proceeding from a data model (Barth et al., 2020), limited predictive performance on out-of-sample data (Zhong et al., 2021), and challenges related to multicollinearity (Chan et al., 2022). In contrast, machine learning offers a data-driven alternative capable (Cheng et al., 2021). By leveraging its superior predictive capabilities (Lee and Kang, 2024), machine learning can model complex relationships among multi-level factors influencing PA and SP without proceeding from a data model (Zhou et al., 2019) and while mitigating risks such as overfitting and multicollinearity (Gosztonyi, 2023; Desai et al., 2020). Though the black box nature prevalent in most machine learning algorithms is yet to result in limitations on the depth to which interpretations about mechanisms could be pursued, with the algorithm “black box” meaning that “users cannot understand the process due to either being information proprietary or too complex to understand” (Shin and Park, 2019) (meaning inputs and output are understandable to user, while not comprehending what's in between), these hitches have now been overcome by interpretative algorithms (Guidotti et al., 2018). Permutation Feature Importance (PFI), Partial Dependence Plot (PDP), for example, improves interpretability for decision-making, output, or result (Hassija et al., 2024). As a result, machine learning is reshaping methodological paradigms in sport science research (Chmait and Westerbeek, 2021). Recent applications include analyses of college students' exercise behaviors (Liu et al., 2023), prediction of user participation in e-sports (Khir et al., 2024), and monitoring of athletic injuries and illnesses (de Leeuw et al., 2022), among other emerging domains.

In summary, promoting adult PA and SP during the COVID-19 pandemic is the result of the combined effect of multi-level factors. While scholars have achieved certain results in deeply exploring the various reasons for adult PA/SP and seeking corresponding promotion strategies, the focus on related variables remains fragmented, often concentrating either on the individual or the environment. This presents a clear risk of viewing the problem in isolation and unilaterally. The Socio-ecological Model, as a continuously evolving theoretical framework, has been empirically validated by numerous international studies and is recognized as an effective approach for systematically analyzing the determinants of PA and SP (Zhang and Solmon, 2013). Furthermore, although the Socio-ecological Model emphasizes predicting individual exercise behavior from different perspectives (Yang et al., 2014) and accounts for the complexity and multidimensional co-action of factors promoting population PA/SP, utilizing traditional statistical models based on previous literature would face limitations such as restricted predictive accuracy and the influence of multicollinearity, making it difficult to achieve the integration of these disparate influencing factors. Therefore, the first objective of this study is to apply the Socio-ecological Model to explore the determinants of PA and SP during the COVID-19 pandemic across four levels: individual characteristics, individual behaviors, interpersonal relationships, and community environment. The second objective is, based on this multilevel analytical foundation, to adopt interpretable machine learning models to further clarify the factors relevant to specific behaviors like promoting population PA/SP, precisely identifying and exploring the common and distinct factors of adult PA and SP during the pandemic to bridge the theory-practice gap and enrich the Socio-ecological Model theory while providing a reference for public health policy formulation.

## 2 Materials and methods

### 2.1 Study design

The quantitative, cross-sectional secondary data analysis was adopted for conducting this analysis, based on the Chinese General Social Survey (CGSS2021 Data Set), to understand its applicability in identifying data-driven, multiple-level determinants for adult PA and SP. Using Socio-ecological Models for constructing its underlying frameworks for referencing, a comprehensive model was built to incorporate 45 factors, such that PA/SP could be determined based on individual characteristics, individual behavior, interpersonal relations, and community environment. Related to preprocessing, while imputing missing values, the “MissForest” algorithm was primarily used, which was then followed by creating binary categorical PA and SP outcomes, with a final sample size for analysis to be 2,717 adult participants. During the data analysis process, the data was first divided into training and testing data in an 8:2 ratio. On the training data, there was a 5-fold cross-validation grid search for eight major machine learning algorithms (Random Forest, XGBoost, etc.), to reveal their optimal parameters. Subsequent analysis, on testing data, assessed model performance based on criteria such as accuracy (ACC), F1 score, and the area under the ROC curve (AUC), to finally choose the optimal predictive model for PA and SP. Using these optimized models, Permutation Feature Importance (PFI) analysis was carried out to establish importance ranks for all factors. These factors were then successively included into these optimized models, in decreasing orders of their importance, with identification of the number of key factors based on identification of the “inflection point” on AUC curves. Finally, to enable an in-depth examination into the specific directions of influence and non-linear patterns exerted by key factors, there was an analysis through Partial Dependence Plots (PDP).

### 2.2 Socio-ecological model

The Socio-ecological Model integrates biological and sociological perspectives in examining individual health behaviors and has been extensively applied in PA and health promotion (Wang et al., 2024). Rather than advocating single-level interventions, this model advocates for multi-level, synergistic interventions to achieve long-term and efficacious behavior change. It provides a comprehensive framework for PA comprehension and promotion. According to this theory, the present study constructs a multi-level model of SP and PA determinants among adults.

**Figure 1:**
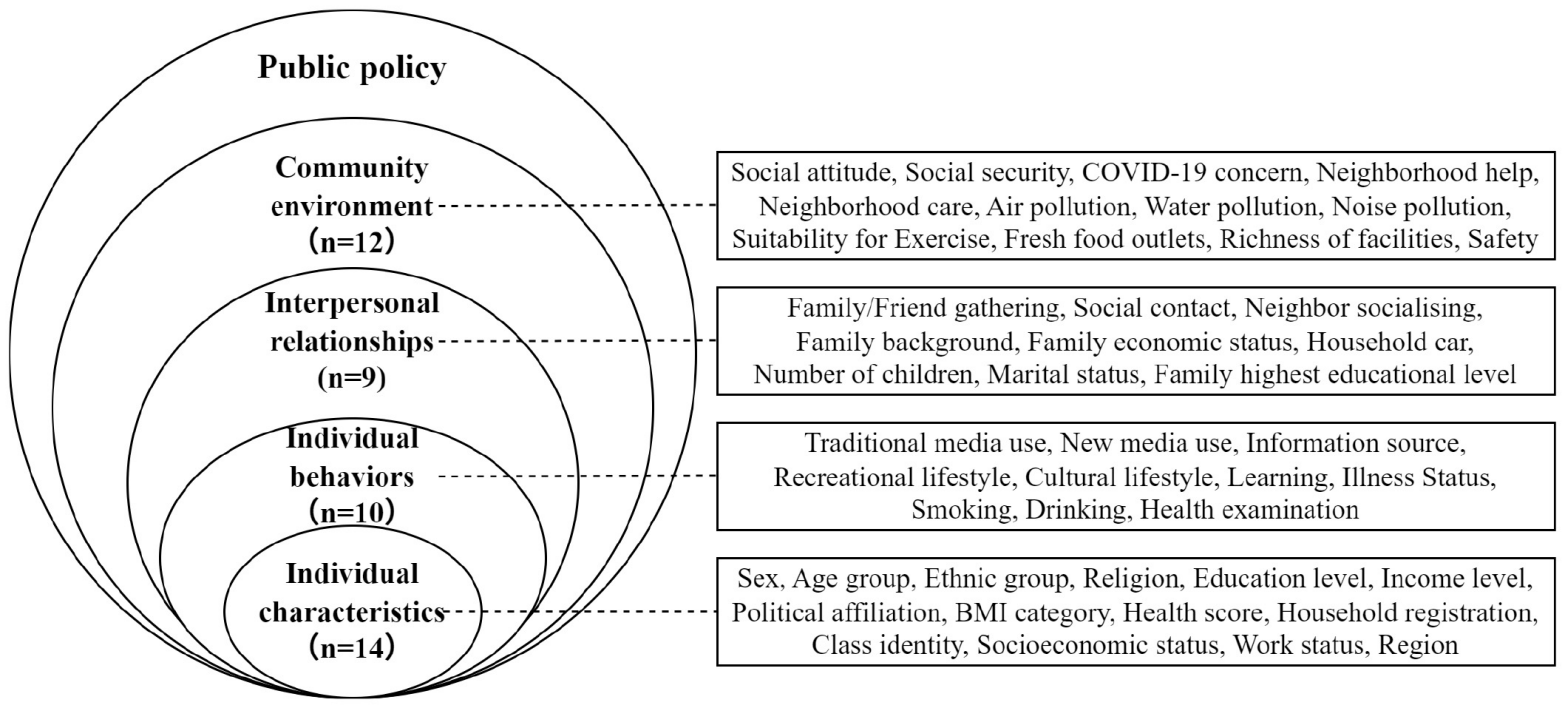
A Socio-ecological Model of factors influencing physical activity and sports participation.

The Socio-ecological Model is a dynamic non-prescriptive one, i.e., it is not strictly following one particular theoretical tradition, and it is widely employed to synthesize published evidence on PA and SP determinants (Sallis et al., 2015). Although the multi-level of the model has varied over time from three to five levels and back again, for the most part, the literature agrees that the model consists of five overarching levels: individual, interpersonal, organization, community, and policy (McLeroy et al., 1988). For the current study, four of the levels are examined (Figure 1): individual characteristics, individual behaviors, interpersonal relationships, and the community environment. The individual characteristics level refers to personal traits, including sociodemographic traits, health status, and working status (Qin et al., 2022). The behavioral level entails lifestyle and health-related behavior (Huang et al., 2024). The level of interpersonal relationships encompasses social relationships and support networks from family, friends, and community members (Xiao et al., 2020). The community environment level consists of broader contextual determinants, including sociocultural norms, natural environment, and built environment (Yi et al., 2016; Zhang et al., 2022). Specifically, this study deals with individual behaviors as being analytically distinct from more general individual qualities to facilitate a greater focus on the final objective of health promotion: encouraging PA and SP through behavior change (Sallis et al., 2006).

### 2.3 Data sources

Data for this analysis came from the 2021 Chinese General Social Survey (CGSS). It was conducted by the National Survey Research Center at Renmin University of China. It was the 14th annual wave, with the 2021CGSS being accomplished after overcoming enormously difficult conditions presented by the COVID-19 outbreak, with assistance from “Surveyor Alliance” universities, taking almost five months to complete, yielding its results to the general public on March 31, 2023 (National Survey Research Centre at Renmin University of China, 2023).

It adopted a complex stratified sample with multiple stages to guarantee the representativeness of its sample on a national level (National Survey Research Centre at Renmin University of China, 2021a). On one hand, its target population comprised all urban and rural households in the 31 provinces, autonomous regions, and municipalities directly under the central government in mainland China, excluding areas in Hong Kong, Macao, and Taiwan. On the other hand, post-stratification weighting was adopted during the process of sample data processing to counteract any problems associated with oversampling bias, thereby improving sample representativeness on a national population level (Bian and Li, 2012). First, in terms of sample assignment, there was initially stratification of the national sample into two major strata, namely “Must-Select Stratum” and “Select-Stratum”. Must-Select Stratum comprised sample households in the top five largest cities' municipal districts: Shanghai, Beijing, Guangzhou, Shenzhen, and Tianjin, identified via factor analysis for variables like GDP, education level, and FDI. Select-Stratum comprised all other urban and rural sample households nationwide, excluding those in Must-Select Stratum. Then, stratified three-stage sampling were employed in each stratum. Must-Select Stratum included stages one, which sampled streets and townships, stage two, which sampled neighborhood committees, to stage three, which sampled final households. Select-Stratum, on the other hand, included 50 sub-strata, with stage one, which sampled districts/county-level cities/counties, stage two, which sampled neighborhood/village committees, to stage three, which sampled final households. Thirdly, in each identified household, one adult aged 18+ was randomly chosen to participate in the survey. To guarantee data quality, rigor, and accuracy in the research, training to guarantee interviewer consistency in conducting these interviews was provided by the survey team, along with mechanisms for on-site surveillance while carrying out surveys. Simultaneously, verification for logical consistency and validation was done on multiple rounds to guarantee maximum authenticity in acquiring these data (Dong et al., 2025).

The 2021 CGSS keeps track of the interaction and dynamic changes in social structure, quality of life, and their inherent mechanisms to connect with each other (National Survey Research Centre at Renmin University of China, 2021b). The topical classification system for CGSS centers on social structure (using positional and relational techniques, covering social stratification, organizational networks, interpersonal relations, etc.), while also emphasizing quality of life, which embraces five dimensions: health, population, psychology, socioeconomic status, and politics/community. More specifically, social structure and quality of life, insofar as their inherent mechanisms are concerned, cover individual cognition, interpersonal networks, organizational resource allocation (family, community, workplace), and institutional rules. It matches ideally with the perspective adopted in the current study, which relies on the Socio-ecological Model to examine multiple levels involved in PA and SP. Criteria for participants to be included in the survey: participants had to be members of their families aged 18 and above, living in the 31 provinces of mainland China, and with the capacity to understand the contents of the questionnaire.

The sampling design in this analysis conforms to the complex, multi-stage stratified probability sampling method adopted in CGSS. It is worth highlighting, however, that in the 2021 survey, the East Asian Social Survey (EASS) Health Module was completed randomly by one-third of those interviewed, containing information on issues such as health condition, healthcare, social trusts, and concerns about aging. It must be noted that such information is not normally captured in other surveys, offering an enriched data platform to examine health behavior among adult populations during these pandemic years. A total sample size of 8,148 valid samples was collected in the entire country in 2021. Owing to the fact that this research relies on variables from EASS Health Module-variables for individual behavior (illness status, smoking, drinking, and health examination), and variables for community environment (COVID-19 concerns, neighborhood help, neighborhood care, air pollution, water pollution, noise pollution, suitability for exercise, fresh food outlets, richness of facilities, and safety) to be studied, given the fact that EASS Health Module has been filled out by only one-third of the randomly selected subjects, a total of 5,341 subjects who did not fill out EASS Health Module were excluded from analysis. Hence, only 2,717 were included in analyzing crucial influencing factors. Subsequently, all responses to variables have been coded uniformly in numeric values. Simultaneously, “not applicable”, “do not know”, and “refusal to answer” response types have also been changed to missing values. Eventually, the missing data percentage varied from 0.1% to 9.1%, with most missing data contributing to factors like “income level”, “Family highest educational level”, and “Family background”, i.e., having 9.1%, 8.6%, and 4.3% in respective orders. Missing value for other variables varied from 0% to 3%. Data missingness is a frequently encountered and increasingly recognized phenomenon (Fernando et al., 2021), hence, for this research, the algorithm used for handling missing values in the data was the non-parametric algorithm “MissForest”, which is based on the random forest algorithm (Stekhoven and Bühlmann, 2012). The reason for choosing “MissForest” was its accuracy, in addition to not needing assumptions to be made on data distribution. Missing values in continuous variables obtained from “MissForest” were then rounded to the nearest integer then used for analysis.

### 2.4 Ethics approval

The Chinese General Social Survey (CGSS), which is the earliest national representative continuous survey project undertaken by an academic institution in mainland China, strictly adhered to the general ethical principles outlined in the Declaration of Helsinki. Research ethics approval was overseen by the Institutional Review Board of Renmin University of China and the Ethics Committee of Hong Kong University of Science and Technology. All participants gave their informed consent before being included in conducting the survey. We received authorization to use the publicly accessible CGSS data. Owing to its administrative nature, which is anonymous to its subject matter, with no identifiable information in its database, its website is available at http://cgss.ruc.edu.cn/. Hence, no ethics approvals were needed for conducting this research.

### 2.5 Measurement of variables

#### 2.5.1 Outcome variables

PA and SP will be the outcome variables in this study. PA can be described as any bodily movement requiring skeletal muscle contraction above the resting level (Caspersen et al., 1985). This has been adopted by the WHO (Waxman, 2004). The reason behind the adoption of this particular description is that PA comprises various forms of activity (Gauthier et al., 2012). SP, although correlated with PA, is actually different from it. It is generally theoretically assumed to be a subset of PA, with planned, structured, and repetitive efforts aimed at improving or maintaining physical fitness (Caspersen et al., 1985). It was specifically defined for the first time by Kenyon in (Kenyon, 1969), taking into consideration several aspects such as cognitive, affective, emotional, and physical actions. It basically suggests intentional sporting activity behavior undertaken by means of physical exercise and recreational activity to achieve improved physical and mental health, enhanced cultural fulfillment, and enhanced social interaction (Yanmin et al., 2025). It consists of two types (Kan and Xie, 2024): Direct participation, in which the person actually participates in physical exercise, while indirect participation entails other means such as being a “spectator/consumer of sports action” (Guo et al., 2024). The current research concentrates on “direct participation”, as it provides better indicators for examining the drivers of structured physical exercise during the pandemic. It conceptually connects with planned, structured, and repetitive exercise programs for health promotion.

The health outcomes being achieved through planned, structured, and repetitive sporting activity have different qualitative and quantitative outcomes from those being achieved through unstructured PA (Eather et al., 2023; Eime et al., 2015). It is hereby understood that in the context of the present analysis, PA and SP as outcome variables for comparative analysis. They capture complementary aspects of health-promoting behavior: an individual may accumulate substantial PA through incidental daily behaviors without engaging in organized sport (Malm et al., 2019). PA refers to the assessment of whether it meets absolute health guidelines for disease prevention and physiological health. On the other hand, SP offers a broader scope of considerations for health integration. This comparative analysis offers an opportunity to facilitate evidence-based policy development for both domains (Volf et al., 2022).

The PA variable was operationalized based on two dimensions: “Daily PA” and “Moderate-to-Vigorous PA (MVPA)” (Yuki et al., 2019). The question “How long do you walk on a typical weekday?” was used to measure Daily PA (Liu et al., 2024b). The question “How many hours per week do you engage in physical activities that make you breathe faster than usual?” was used to measure MVPA (Zou and Cui, 2024). These questions, which have high levels of validation for physically active research (Milton et al., 2013), have also been utilized in the International PA Questionnaire (IPAQ) (van der Ploeg et al., 2010; Cleland et al., 2018). Researchers have employed these questionnaire data from CGSS to examine problems concerning PA (Liu et al., 2024b; Zou and Cui, 2024), thereby validating its reliability and appropriateness in CGSS. Total weekly PA was calculated by multiplying “Daily PA” by 5, in addition to summing the weekly “MVPA” duration. To be precise, the multiplication factor of 5 was only used for the “Daily PA” so as to estimate the weekly volume from the typical weekday data. The “MVPA” variable was not multiplied, since the item in the survey measured directly the overall duration per week. Thus, the total weekly PA was defined by the formula: Total Weekly PA = (“Daily PA” × 5) + “MVPA”. This operationalization acknowledges that PA health effects are garnered based on activity intensity levels (Murphy et al., 2019). Walking is the primary mode of Daily PA for adults, being the most commonly reported leisure-time PA and contributing significantly to the weekly total PA (Audrey et al., 2014). On the other hand, “MVPA” focuses on PA requiring effort to increase cardiovascular activity (MacIntosh et al., 2021). Following guidelines set out in “PA and Sedentary Behavior Guidelines for Chinese People in 2021” (PASBG 2021) (Chen et al., 2022), in which it is stipulated to have on average 150 to 300 minutes per week with “moderate-intensity PA”. This threshold is fully consistent with the 2020 World Health Organization guidelines (Bull et al., 2020) and the recommendations of authoritative bodies such as the American Heart Association (Strath et al., 2013). The point of 150 minutes, identified in numerous studies, is just enough to provide substantial health gains (Steenkamp et al., 2022; Alshehri, 2024). Within studies conducted on similar contexts, those reaching 150 minutes or more are considered to be “physically active”, while those not reaching 150 minutes are “physically inactive” (Almeida et al., 2014). Thus, in the present study, PA levels were categorized as a dichotomous variable. Participants who engaged in less than 150 minutes of PA per week were coded as “not meeting standard” and assigned a value of 0. Participants who engaged in 150 minutes or more per week were coded as “meeting standard” and assigned a value of 1.

The SP variable was measured with the question: “Over the past year, have you regularly participated in sports activities during your leisure time?” (Chao et al., 2025). Such a self-reported method for SP measurement has been utilized in many studies (Kan and Xie, 2024; Liu and Zhong, 2023) on a large epidemiological scale for long-term trend analysis (Zhang et al., 2025). Researchers carrying out studies on SP with regard to CGSS 2021 have also utilized such an item to measure SP (Wu, 2022; Zhang and Zhang, 2021), and given its extensive use and high reliability, the item's reliability and validity are established within the CGSS context (Zhang et al., 2024b). Most studies have described SP as a dichotomous classification variable (Eime et al., 2013). SP is viewed in terms of a continuous health behavior, requiring data to demonstrate temporally continuous consistency (Yu et al., 2025). A threshold of “Several times a month” is typically deemed sufficient to establish behavioral consistency. On the other hand, “Several times a year or less” reflects fragmented, occasional participation, which fails to constitute a pattern of regular SP (Yu and Xu, 2021). Accordingly, after literature consultation (Peng and Xu, 2024), “Several times a year or less” and “Never” were coded as “non-participation” and assigned a value of 0. Conversely, “Every day”, “Several times a week”, and “Several times a month” were coded as “participation” and assigned a value of 1.

However, we do understand that in dichotomizing these outcome variables (150 minutes/week, “regular participation”), we have obviously lost some variation in their broader complexity in their original forms. Nevertheless, this method enables us to clearly identify the factors that prompt adults to cross the minimal participation threshold for healthy behavior during the pandemic. This is particularly crucial for formulating targeted, resource-efficient public health intervention strategies in the post-pandemic era.

#### 2.5.2 Factors

The selection of the factors was informed by three main criteria: (1) empirical support from literature through past studies with significant associations with PA or SP; (2) theoretical alignment with the Socio-ecological Model; (3) presence in the CGSS2021 dataset. According to these criteria, a total of 45 factors were identified, covering four levels of the Socio-ecological Model. More specifically, 14 factors relate to individual characteristics (e.g., sex, age group, and education level), 10 to individual behaviors (e.g., media use), 9 to interpersonal relations (e.g., social contact and family economic status), and 12 to the community environment level (e.g., built environment). Multi-level classification of the factors by the Socio-ecological Model is depicted in Figure 1. The survey items and the definitions of the measures of all the factors at the different levels are provided in the Supplementary Table 1.

### 2.6 Interpretable machine learning approach

Eight well-known machine learning algorithms were implemented here: Logistic Regression (LR) (Cox, 1958), Support Vector Machine (SVM) (Cortes and Vapnik, 1995), Decision Tree (DT) (Quinlan, 1986), Random Forest (RF) (Breiman, 2001), Adaptive Boosting (AdaBoost) (Freund and Schapire, 1995), Gradient Boosting Decision Tree (GBDT) (Friedman, 2001), eXtreme Gradient Boosting (XGBoost) (Chen and Guestrin, 2016), and Light Gradient Boosting Machine (LGBM) (Ke et al., 2017). These algorithms have been selected because of their proven capabilities in prediction, stability, and are popular in the literature on sports sciences (Fu et al., 2024; Barth et al., 2020; Zhou et al., 2019; Lee and Kang, 2024; Chmait and Westerbeek, 2021; Liu et al., 2023; Zhao et al., 2023).

To create an efficient workflow, the data (2,717 samples in total) was divided into a training set (consisting of 80% samples) and test data (consisting of 20% samples). For dividing, scikit-learn's “train_test_split” function was utilized, with “stratify=y” for maintaining class representation in output, while “random_state = 42” was fixed for reproducibility. Hyperparameter tuning was methodically done solely on the training set to identify the best set for each of these eight algorithms with regard to both outcome variables. A coarse grid search for primary model parameters was first undertaken with scikit-learn's “GridSearchCV” package (Liashchynskyi and Liashchynskyi, 2019) with 5-fold stratified cross-validation (StratifiedKFold, n_splits=5, shuffle=True, random_state=42) to optimize for performance on “roc_auc” (the specific search spaces are detailed in Supplementary Table 2). Subsequent to these, further refinement in model parameters via manual testing based on model performance measures was done. In cases of overfitting, we adjusted key regularization parameters. Conversely, when underfitting occurred, we explored increasing model complexity. Optimal model parameters for each algorithm are listed in Supplementary Table 2. For the purpose of model comparison in the final analysis, the model with optimal hyperparameters (best_estimator_) based on grid search results was tested on unseen data in the test set. Evaluation for performance was carried out based on three common criteria: accuracy (ACC), F1 score (F1), and the area under the ROC curve (AUC). While ACC and F1 are calculated based on the classification output for the test set (y_pred), AUC is calculated based on probabilities (y_prob), which are generated from model predictions via “predict_proba” or “decision_function” for SVM models.

For determining the factors having the most influence, the optimal predictive model was adopted as the analytical basis, and the PFI approach (Fisher et al., 2019) was used to express the relative importance of every factor in quantitative terms. The process involved the following three key steps: (1) the factors having positive PFI were progressively added to the optimal model in decreasing importance; (2) after each additional step, the model was tested by the AUC score to evaluate the marginal effect of the newly added factor on the predictive ability; (3) a line graph was plotted to show the change in AUC scores as the number of added factors increased and represent the direction of model improvement. The point where the curve starts to level off was determined as the “inflection point”, denoting the point where incorporating additional factors provides decreasing returns. The number of factors at the inflection point was determined as the subset of the most significant factors responsible for the prediction of the target outcome. Furthermore, for better comprehension of the impact of the key factors on the target outcomes, the PDP were plotted and used to interpret the direction of the impacts. The complete analyses have been conducted through Python (version 3.11), as well as its packages, including scikit-learn (version 1.2.2), XGBoost (version 2.1.4), and LightGBM (version 4.6.0). The whole code has been made available on GitHub: https://github.com/Aikecode/PA-SP-ML.

## 3 Results

### 3.1 Descriptive statistics

A total of 2,717 participants took part in this research. Table 1 shows the descriptive statistics for the variables in this research. On the dimensions of the outcome variables, 1,461 participants (53.77%) showed their engagement in SP, while 1,866 participants (68.68%) met the standard for PA. Respondents generally showed an almost balanced composition in terms of their sex, with 1,228 participants (45.2%) from the Male gender, while those from the female sex comprised 1,489 participants (54.8%). On the dimensions of the participants' classification based on their “Age group”, 929 participants (34.19%) belong to “Young adult”, while 778 participants (28.63%) belong to “Middle-aged adult”. Lastly, participants from “Older adult” composed 1,010 participants (37.17%). Furthermore, 2,482 participants (91.35%) were categorized as Without Religious Belief. By Educational level, 909 participants (33.46%) have Grade school or below, 764 (28.12%) have Junior high school, 552 (20.32%) have Senior high school, while 492 (18.11%) have Junior College or above. Regarding the BMI group, most belong to Normal weight, with 1,480 participants (54.47%), followed by Overweight with 757 (27.86%), Obese with 255 (9.39%), while 225 (8.28%) belong to the Underweight group. Geographically, most participants lived in the Eastern Region, specifically 1,098 participants (40.41%), followed by the Central Region with 913 (33.60%), while 706 participants (25.98%) lived in the Western Region. Moreover, more than half of all participants possessed rural household registration. Examining the household context, the largest proportion of participants rated their Family economic status as Average (1,413 individuals, 52.01%). By the Family's highest Educational level, most participants have Grade school or below, specifically 1,860 participants (68.46%). Finally, regarding Marital status, 704 participants (25.91%) reported no partner, while 2,013 (74.09%) were partnered.

**Table 1:** Descriptive statistics of variables (*n* = 2,717).

**Variable name**	**Level**	**Count**	**Percentage, %**	**Mean (SD)**
**Outcome variables**				
Sports participation				0.54 (0.50)
	Non-participation	1256	46.23	
	Participation	1461	53.77	
Physical activity				0.69 (0.46)
	Not meeting standard	851	31.32	
	Meeting standard	1866	68.68	
**Individual characteristics**				
Sex				1.55 (0.50)
	Male	1228	45.20	
	Female	1489	54.80	
Age group				2.03 (0.84)
	Young adults	929	34.19	
	Middle-aged adults	778	28.63	
	Older adults	1010	37.17	
Ethnic group				1.08 (0.26)
	Han	2512	92.45	
	Ethnic minority	205	7.55	
Religion				1.09 (0.28)
	Without religious belief	2482	91.35	
	With religious belief	235	8.65	
Education level				2.25 (1.12)
	Grade school or below	909	33.46	
	Junior high school	764	28.12	
	Senior high school	552	20.32	
	Junior College or above	492	18.11	
Income level				2.44 (1.09)
	Lowest	696	25.62	
	Lower middle	718	26.43	
	Upper middle	709	26.09	
	Highest	594	21.86	
Political affiliation				1.12 (0.33)
	Non-party member	2386	87.82	
	Party member	331	12.18	
BMI category				2.38 (0.77)
	Underweight	225	8.28	
	Normal weight	1480	54.47	
	Overweight	757	27.86	
	Obese	255	9.39	
Health score				3.78 (0.92)
Household registration				1.70 (0.46)
	Urban	827	30.44	
	Rural	1890	69.56	
Class identity				4.30 (1.82)
Socioeconomic status				2.29 (0.89)
Work status				1.51 (0.50)
	Unemployed	1336	49.17	
	Employed	1381	50.83	
Region				1.86 (0.80)
	Eastern	1098	40.41	
	Central	913	33.60	
	Western	706	25.98	
**Individual behaviors**				
Traditional media use				2.11 (0.69)
New media use				2.64 (1.24)
Information source				1.62 (0.49)
	Traditional media	1044	38.42	
	New media	1673	61.58	
Recreational lifestyle				2.87 (0.75)
Cultural lifestyle				1.67 (0.69)
Learning				2.12 (1.21)
Illness Status				1.38 (0.48)
	Without chronic disease	1690	62.20	
	With chronic disease	1027	37.80	
Smoking				1.55 (0.84)
Drinking				1.82 (1.24)
Health examination				2.03 (0.77)
	No	763	28.08	
	Irregular	1101	40.52	
	Regular	853	31.39	
**Interpersonal relationships**				
Family/Friend gathering				2.18 (0.76)
Social contact				2.66 (1.13)
Neighbor socialising				3.76 (2.24)
Family background				3.29 (1.92)
Family economic status				2.61 (0.75)
	Well below average	207	7.62	
	Below average	883	32.50	
	Average	1413	52.01	
	Above average	201	7.40	
	Well above average	13	0.48	
Household car				1.44 (0.50)
	Without car	1533	56.42	
	With car	1184	43.58	
Number of children				2.37 (0.73)
	No children	409	15.05	
	Only child	883	32.50	
	Multiple children	1425	52.45	
Marital status				1.74 (0.44)
	No partner	704	25.91	
	Partnered	2013	74.09	
Family highest educational level				1.50 (0.84)
	Grade school or below	1860	68.46	
	Junior high school	468	17.22	
	Senior high school	276	10.16	
	Junior College or above	113	4.16	
**Community environment**				
Social attitude				3.54 (0.63)
Social security				1.97 (0.16)
	Non-participation	75	2.76	
	Participation	2642	97.24	
COVID19 concern				2.50 (1.06)
	Not at all concerned	578	21.27	
	Not very concerned	810	29.81	
	Somewhat concerned	730	26.87	
	Very concerned	599	22.05	
Neighborhood help				4.01 (0.80)
Neighborhood care				3.94 (0.87)
Air pollution				1.95 (0.78)
Water pollution				1.96 (0.80)
Noise pollution				1.97 (0.83)
Suitability for Exercise				3.82 (1.01)
Fresh food outlets				4.10 (0.83)
Richness of facilities				3.18 (1.25)
Safety				4.19 (0.71)

### 3.2 Model comparison

During the comparative analysis stage, we assessed their performance based on three major criteria: Accuracy (ACC), Area Under the ROC Curve (AUC), and F1 Score. These criteria were selected to obtain a complete performance analysis for each classification model on these three crucial dimensions: overall accuracy, classification capacity, and optimal balance between precision and recall rates, which are especially vital in health behavior data (Lee and Kang, 2024). From Table 2, for PA classification, the RF model possessed the highest AUC value of 0.613, signifying its highest classification accuracy in segregating participants meeting the general PA guidelines from those violating them. Moreover, its high ACC value of 0.697 with an optimal F1 Score value of 0.816 indicated its highest overall accuracy, stability, and optimal classification capability in different settings. Given that RF is an ensemble method and is relatively effective in capturing the complex, nonlinear interactions among multiple factors determining PA (Ellis et al., 2014), it was identified as the optimal model for PA analysis.

**Table 2:** Comparison of eight mainstream machine learning algorithms.

	**Physical activity**			**Sports participation**		
	**ACC**	**AUC**	**F1 score**	**ACC**	**AUC**	**F1 score**
LR	0.695	0.601	0.814	0.704	0.770	0.721
SVM	0.688	0.598	0.815	0.717	0.778	0.734
DT	0.695	0.568	0.813	0.675	0.739	0.695
RF	0.697	0.613	0.816	0.711	0.777	0.732
GBDT	0.697	0.594	0.817	0.710	0.781	0.727
AdaBoost	0.695	0.604	0.813	0.713	0.772	0.731
XGBoost	0.697	0.605	0.816	0.719	0.772	0.734
LGBM	0.695	0.601	0.816	0.715	0.773	0.732

ACC, Accuracy; AUC, Area Under the ROC Curve; LR, Logistic Regression; SVM, Support Vector Machine; DT, Decision Tree; RF, Random Forest; GBDT, Gradient Boosting Decision Tree; AdaBoost, Adaptive Boosting; XGBoost, eXtreme Gradient Boosting Model; LGBM, Light Gradient Boosting Machine.

Concerning the predictions for SP, XGBoost possessed the highest value for ACC (0.719) and F1 (0.734), indicating its high accuracy in identifying participation predictions, along with an optimal balance between precision and recall. On the other hand, its AUC value of 0.772 indicated its highly efficient discriminatory power. XGBoost is an efficient algorithm for gradient boosting, which has demonstrated its high accuracy and efficiency in identifying the probability of regular SP (Antolini et al., 2025). Hence, XGBoost was chosen for analysis concerning SP.

### 3.3 Key factors influencing physical activity and sports participation

#### 3.3.1 Identification of key influencing factors

A total of 10 and 12 key factors were identified as significantly influencing PA and SP, respectively. These numbers were determined by the point at which model performance no longer substantially improved with the inclusion of additional factors, as identified by the inflection point in the AUC curve. For context, PFI values were calculated for all factors in the optimal models. A positive PFI value indicates a meaningful contribution to model performance. Results showed that 30 factors had positive PFI values in the optimal RF model predicting PA, while 29 factors showed positive PFI values in the optimal XGBoost model predicting SP.

**Figure 2:**
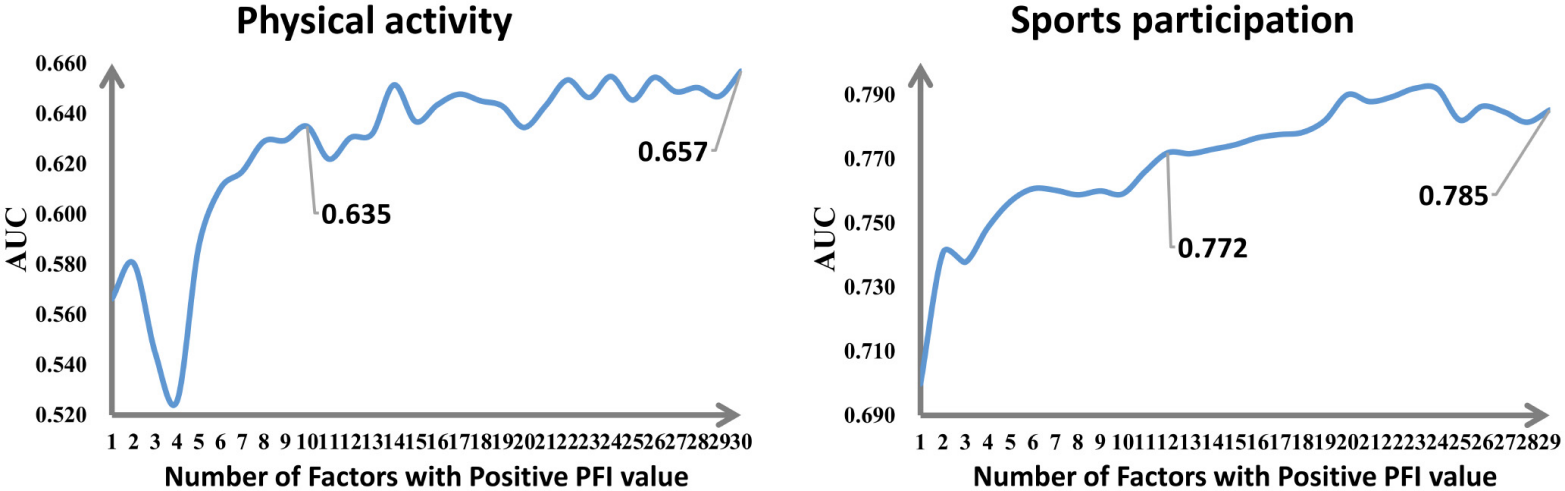
Line plot to determine the number of key factors based on the AUC scores.

**Figure 3:**
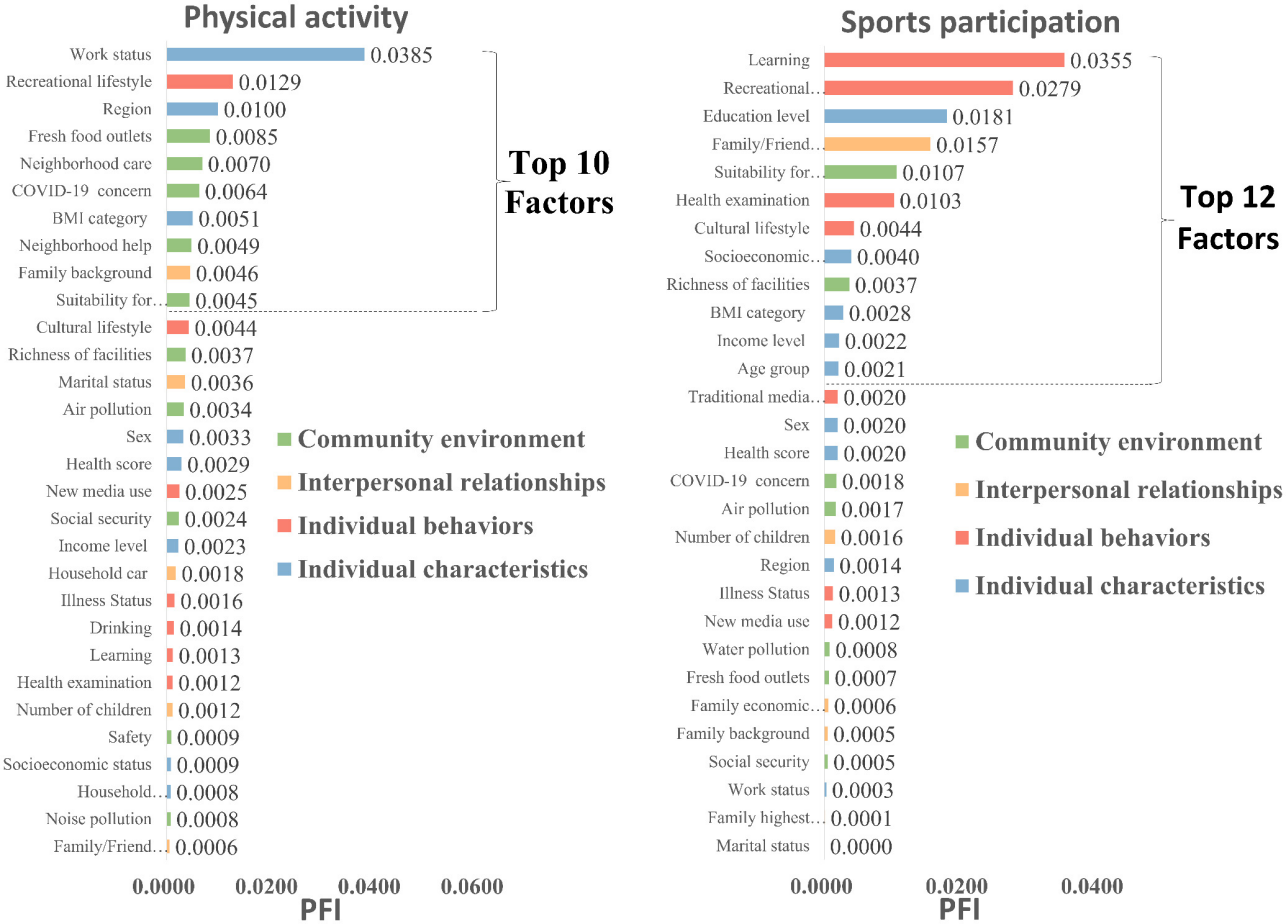
Permutation feature importance values for factors of physical activity and sports participation.

As shown in Figure 2, the AUC of the optimal model for PA prediction had reached 0.657 when all the factors with positive PFI had been used. This was superior to the model with all the 45 initial factors. When the top 10 ranked factors had been used individually, the model had an AUC of 0.635 and represented 96.65% (0.635/0.657) of the performance when all the 30 positive contributing factors had been used. Also for SP, the optimal model had an AUC of 0.785 on utilizing all the 29 factors with positive PFI, which was better than the full model including all the 45 factors. On utilizing the top 12 ranked factors, the AUC was still good at 0.772, which was equivalent to 98.3% (0.772/0.785) of the full model's performance. These outcomes mean that not all original factors contributed constructively to the model because the full range of 45 factors was found to be redundant. The list of the factors with positive PFI associated with PA and SP is presented in Figure 3.

#### 3.3.2 Key factors influencing physical activity

**Figure 4:**
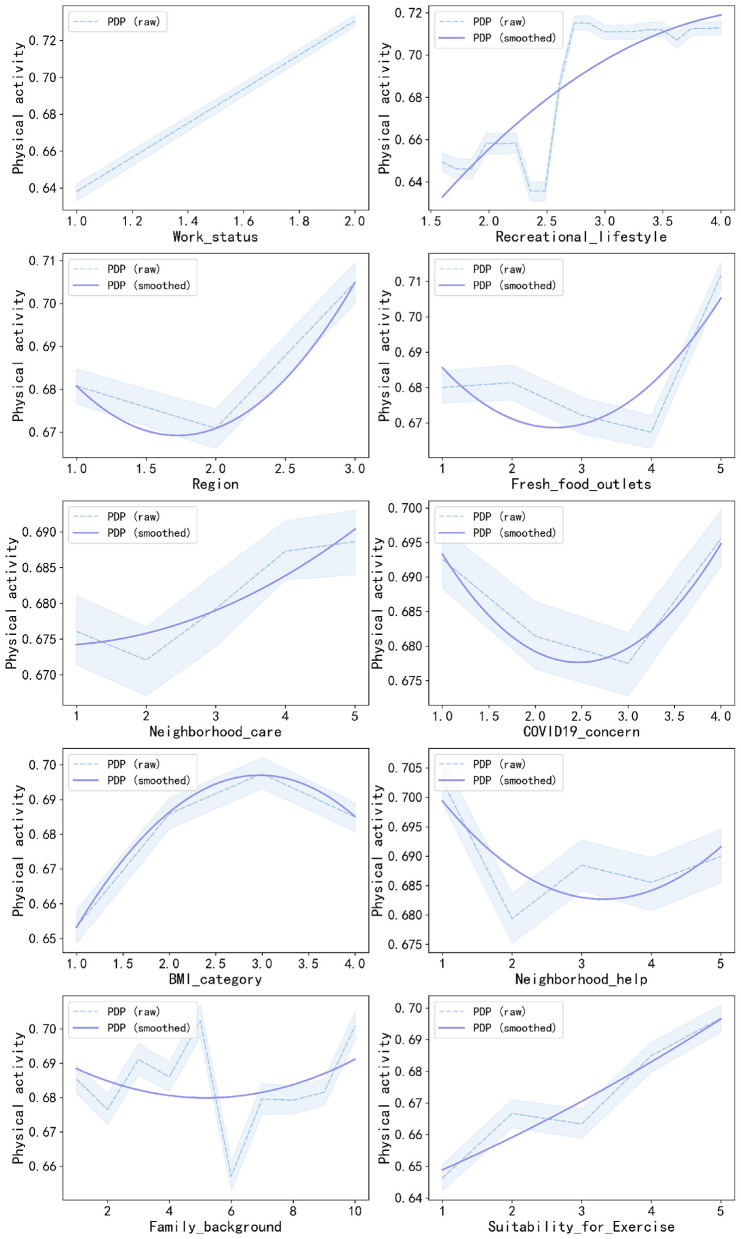
Partial dependence plots of key factors associated with physical activity.

Figure 4 further illustrates the effects of the top 10 key factors associated with PA. Table 3 supplements the graphs with the detailed predicted probabilities used in the PDP. At the individual characteristics level, working adults (Rank 1) have a higher predicted probability of meeting the standard of PA at probabilities of 0.730 compared to their unemployed counterparts. Similarly, adults from the western region (Rank 3) are likely to achieve the same at a probability of 0.705 compared to those from the remaining regions. Adults with a normal or overweight BMI (Rank 7) also have a higher likelihood of the desired standard at probabilities of 0.686 and 0.697, respectively. At the individual behavioral level, the adults who ranked the highest in recreational lifestyle (Rank 2) showed a probability of 0.713 to comply with the standards. At the interpersonal relationships level, the adults who came from a relatively privileged background (Rank 9) had a higher likelihood of showing the correct PA behavior. The maximum probability of this behavior was 0.702. At the community environment level, the probability of achieving the standard of PA had the highest value of 0.712 at a point when the availability of fresh food outlets (Rank 4) was highest. The highest level of neighborhood care (Rank 5) had a probability of 0.689, while neighborhood help (Rank 8) also had a positive association. Furthermore, the greater the level of suitability of exercise (Rank 10), the larger the probability of achieving the standard, from 0.646 to 0.697. In addition, adults who chose “not at all worried” or “very worried” about COVID-19 infection (Rank 6) demonstrated a higher possibility of being involved in enough levels of physical activities at probabilities of 0.693 and 0.696, respectively.

**Table 3:** Predicted probability of physical activity across levels of key factors.

**Key factor**	**1**	**2**	**3**	**4**	**5**
Work status	0.638	0.730	-	-	-
Recreational lifestyle	-	0.658	0.711	0.713	-
Region	0.681	0.671	0.705	-	-
Fresh food outlets	0.680	0.681	0.672	0.667	0.712
Neighborhood care	0.676	0.672	0.679	0.687	0.689
COVID-19 concern	0.693	0.681	0.678	0.696	-
BMI category	0.653	0.686	0.697	0.685	-
Neighborhood help	0.703	0.679	0.688	0.686	0.690
Family background	0.685	0.677	0.691	0.686	0.702
Suitability for exercise	0.646	0.667	0.663	0.685	0.697

The values shown inside the cells represent the predicted average probability of meeting the physical activity standard at each factor level obtained from partial dependence plots. The columns labeled 1, 2, 3, 4, and 5 represent the particular values taken by each of these key factors. The symbol “-” represents the lack of a corresponding level associated with the factor.

Starting with a holistic view based on the levels in Socio-ecological Models, the Community environment level accounted for half of the top 10 key factors influencing PA. Simultaneously, based on Figure 3, while interpreting all 30 factors with positive PFI values for PA, it was observed that out of these, again, the highest “Community environment level” factors contributed to PA (*n* = 10) compared to Individual characteristics (*n* = 8), Individual behaviors (*n* = 7), and Interpersonal relationships (*n* = 5). Such environmental dominance is verified in Figure 4 through PDP curves and the data in Table 3, indicating that with improvements in fresh food outlets (Rank 4), neighborhood care (Rank 5), and suitability for exercise (Rank 10), the probability of meeting PA standards significantly increases. This evidence indicates that within the socio-ecological framework, the community environment plays a principal role in shaping PA, highlighting its passive and environmentally dependent nature during the pandemic.

#### 3.3.3 Key factors influencing sports participation

**Figure 5:**
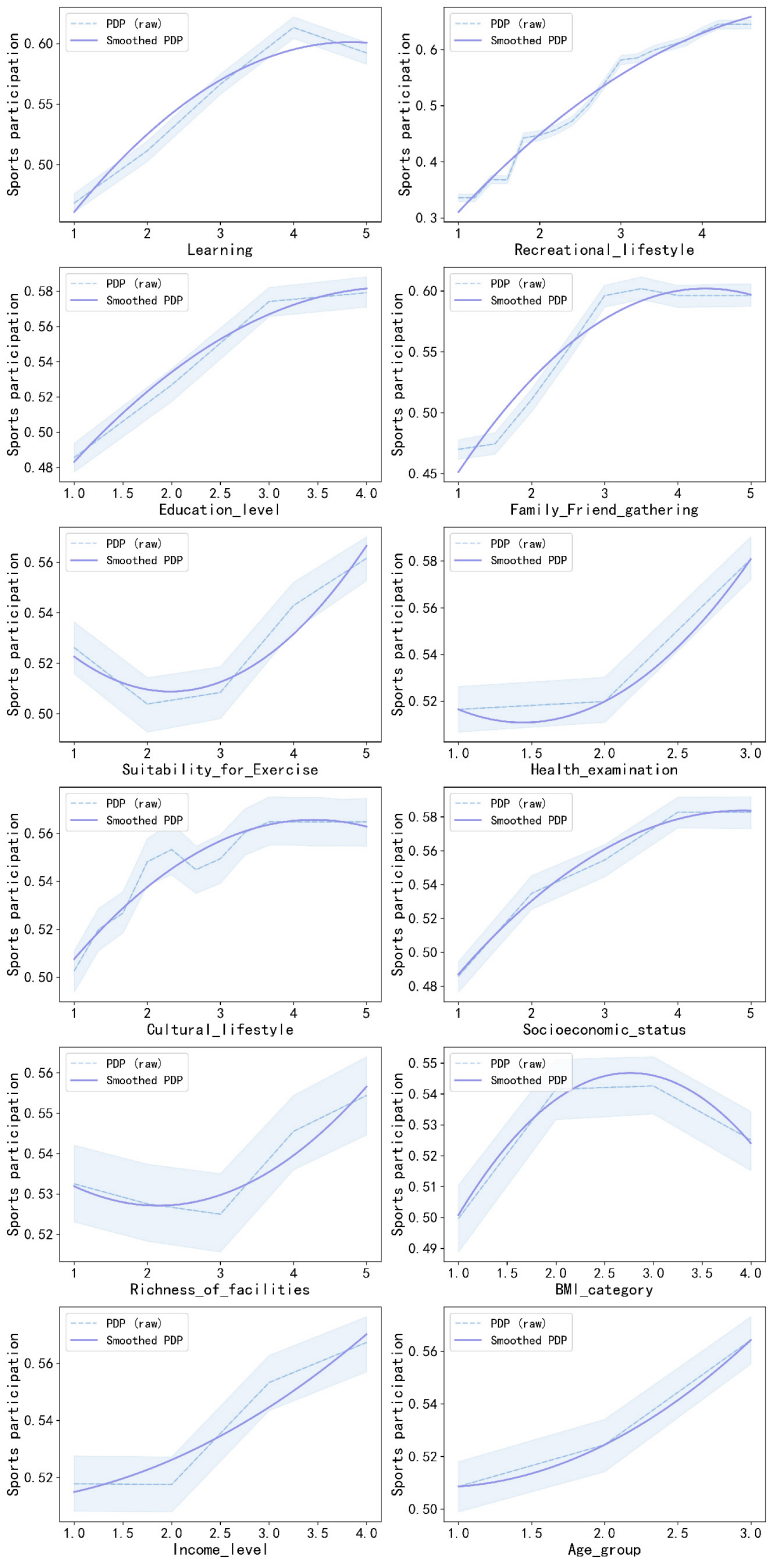
Partial dependence plots of key factors associated with sports participation.

Figure 5 shows the influence of the top 12 key factors of SP. Table 4 supplements the graphs with the detailed predicted probabilities used in the PDP. At the individual characteristics level, participants who had the highest levels of education (Rank 3), socioeconomic status (Rank 8), and income (Rank 11) showed increased probabilities of being involved in sports of 0.579, 0.583, and 0.567, respectively. People who had a value of their BMI classified as normal or overweight (Rank 10) had comparatively higher probabilities of being involved in sports of 0.542 and 0.543. Additionally, participants who belong to the older group (Rank 12) had the highest probability of 0.564. At the individual behavioral level, the group of participants who ranked a high frequency of learning (Rank 1), the highest recreational lifestyle (Rank 2), and the highest level of the cultural lifestyle (Rank 7) demonstrated a high probability of practicing sports at the levels of 0.613, 0.632, and 0.565, respectively. The group of participants who regularly had health examinations (Rank 6) also demonstrated a relatively high probability of practicing sports at the level of 0.581. At the level of interpersonal relationships, the increase in the frequency of gatherings related to family and friends (Rank 4) showed a positive link to SP, causing the probability to increase from 0.470 to 0.596. Lastly, in the community environment, the factors of high suitability for exercise (Rank 5) and the richness of facilities (Rank 9) proved to be positive contributors to participating in active sports at the adult level, since they resulted in the highest probabilities of 0.562 and 0.554, respectively.

**Table 4:** Predicted probability of sports participation across levels of key factors.

**Key Factor**	**1**	**2**	**3**	**4**	**5**
Learning	0.468	0.512	0.566	0.613	0.592
Recreational lifestyle	0.336	0.447	0.581	0.632	-
Education level	0.486	0.527	0.574	0.579	-
Family/friend gatherings	0.470	0.511	0.596	0.596	0.596
Suitability for exercise	0.526	0.504	0.508	0.543	0.562
Health examination	0.516	0.520	0.581	-	-
Cultural lifestyle	0.503	0.548	0.549	0.565	0.565
Socioeconomic status	0.486	0.535	0.555	0.583	0.583
Richness of facilities	0.532	0.528	0.525	0.545	0.554
BMI category	0.500	0.542	0.543	0.525	-
Income level	0.518	0.518	0.553	0.567	-
Age group	0.509	0.524	0.564	-	-

The values shown inside the cells represent the predicted average probability of sports participation at each factor level obtained from partial dependence plots. The columns labeled 1, 2, 3, 4, and 5 represent the particular values taken by each of these key factors. The symbol “-” represents the lack of a corresponding level associated with the factor.

Starting with a holistic view based on the levels in the Socio-ecological Model, factors at the individual level dominate the key determinants for SP. Of these top 12 key factors, five were from Individual characteristics, four from Individual behavior, while fewer came from the levels of Interpersonal relationships (*n* = 1) and Community environment (*n* = 2). This individual-level prominence is also reflected in the 29 factors with positive PFI values (Figure 3). Individual characteristics (*n* = 9) and Individual behaviors (*n* = 7) are numerous, whereas Interpersonal relationships (*n* = 6) and Community environment (*n* = 7) factors are comparatively fewer. It is indicated that SP is heavily influenced by individual features. The PDP curves in Figure 5 and the data in Table 4 provide empirical visualization for this finding. The probability of SP rises significantly with improvements in factors related to socio-cultural and economic capital, such as education level (Rank 3), socioeconomic status (Rank 8), income level (Rank 11), and cultural lifestyle (Rank 7), and with proactive health lifestyles, including learning (Rank 1), recreational lifestyle (Rank 2), and regular health examination (Rank 6). Comprehensively, this evidence indicates that SP behavior is more initiative-driven and linked to the accumulation of an individual's socio-cultural and economic capital.

## 4 Discussion

Habits associated with modern lifestyle, which in itself leads to low levels of PA and also sedentary behavior, have been significantly worsened due to the COVID-19 outbreak. A restricted PA was observed during isolation times, for all ages, due to restrictions on activity. Gyms, pools, or playgrounds, which are meant for sporting activities, in many countries, were shut down, whereas communication via online means for work, recreation, and purchase became important (Shahidi et al., 2020). Within the sports sector, all types of sporting activity were either canceled or postponed, ranging from mass participation events such as marathons to professional sporting competitions, including the Summer Olympic Games (Bok et al., 2020). These scenarios presented several challenges in the promotion of PA, with long-term effects on SP. Notably, there was an emergence of an utmost need to understand the dynamic changes in PA and SP patterns to inform interventions in the sporting management sector (Eime et al., 2022). This study addresses this need directly by applying a Socio-ecological Model and an interpretable machine learning approach. This paper identifies the common and distinct factors influencing adult PA and SP during the pandemic, to facilitate the targeted allocation of intervention resources to key influencing domains.

### 4.1 Common factors influencing physical activity and sports participation

The BMI category is equally important for PA and SP, as it can influence participation through its effects on performance, motivation, or confidence (Zervou et al., 2017). In the present study, the relationship between BMI and both PA and SP during the COVID-19 pandemic was found to be non-linear. One possible explanation is that underweight adults may engage less in PA due to reduced physical fitness levels (Essiet et al., 2017). Furthermore, both underweight status and severe obesity have been associated with increased risk of disability and poorer overall health outcomes (Imai et al., 2008), which may further limit PA engagement in these groups during the COVID-19 pandemic. Conversely, adults with a normal BMI are more likely to maintain regular exercise habits and demonstrate significantly higher levels of PA (Tian et al., 2016). Additionally, overweight adults may be more motivated to engage in PA during the COVID-19 pandemic for the purposes of weight management and health improvement (Kuan et al., 2011), and are often able to achieve sufficient frequency and duration of activity to meet recommended standards (Can et al., 2022).

Recreational lifestyle (Rank 2) have a significant impact on PA and SP among adults. According to a survey study by Jia (2020), participation in recreational activities during the COVID-19 pandemic is commonly viewed as a source of relaxation and personal gratification, and about half the population takes part in the activity as a way of promoting physical well-being. Moreover, those who, as adults receive positive experiences and feelings of satisfaction from a recreational lifestyle are themselves more apt to carry this frame of mind over into PA and SP (Özkan et al., 2021). Meanwhile, the spread of technologies of communicative media and the development of network culture has increasingly imbued SP with entertainment qualities during the COVID-19 pandemic, underscoring the role of recreational lifestyle in influencing behavior (Jia and Ye, 2024). The suitability for exercise (Rank 5) played a stronger role in both PA and SP. This is because better provision of sports facilities is usually associated with an increase in PA and SP (Eime et al., 2017). Moreover, during the COVID-19 pandemic, lack of space and access to sporting facilities are prime deterrents hindering PA and SP by adults (Wang and Wang, 2020).

### 4.2 Distinct factors influencing physical activity and sports participation

Among the distinct factors of PA, work status was the most powerful. Working-age adults during the COVID-19 pandemic have the ability to establish more routine daily lives and travel agendas, which create stable environments conducive to the building of regular PA routines. PA often includes either voluntary or automatic initiation, typically under regularized temporal and environmental routines, as well as repeated behavioral experiences serving as reinforcement (Hagger, 2019). A work-related life style, as a result, supports the acquisition of regular PA habits during the COVID-19 pandemic. Furthermore, employed adults have been found to exhibit a higher level of personal accomplishment and value satisfaction, which can contribute to an optimal psychological profile toward the handling of their health and support PA participation during the COVID-19 pandemic. For example, work satisfaction was found to be positive with the regular participation in PA during the COVID-19 pandemic (Dallmeyer et al., 2023). Region (Rank 3) was another significant factor. During the COVID-19 pandemic, adults living in the western region reported higher levels of PA than those living in the central and eastern regions, as the eastern region was the most severely affected by the pandemic (He and He, 2024). At the level of interpersonal relationships, family background was the sole factor having a significant influence. People belonging to better-off family backgrounds tend to enjoy higher economic resources available to them (Nielsen et al., 2012), thus enhancing access to opportunities and environments for PA during the COVID-19 pandemic. Factors at the community environment level have a significant impact on PA. The availability of fresh food stores during the COVID-19 pandemic prompts higher rates of walking and lower rates of sitting by adults (Liu et al., 2024a). Moreover, the COVID-19 pandemic triggered an outpouring of solidarity among residents of neighborhoods around the world (Terbeck et al., 2023), and an encouraging and friendly neighborhood environment prompts PA and trust by promoting social interaction among residents (Kepper et al., 2019). Furthermore, COVID-19 concern (Rank 6) was associated with the level of PA as well. Those who had been highly worried took up PA as a preventive approach to managing their health as a response to the perceived threat from the disease outbreak (Kercher et al., 2022), whereas those who had never been worried about the issue remained in their established routine of activity without interruption (Mason et al., 2022).

At the individual characteristics level, the distinct factors of adult SP reflect the accumulation of their cultural, economic, and social capital, which typically represent key resources available for access to organized SP (Vandermeerschen et al., 2017). Therefore, the higher socio-economic status population, which had already been motivated to engage in health behaviors before the pandemic, became more aware of the need to maintain and improve their health in the face of behavioral restrictions during the COVID-19 pandemic (Kyan and Takakura, 2022). Another individual characteristic is age group (Rank 12). It may be possible that during the COVID-19 pandemic, the older age group saw SP as a way to connect with friends (visiting each other's homes was not permitted, but going for a walk together was) (Bartlett et al., 2021). Meanwhile, studies suggest that younger people were more affected by the closure of sports facilities and organized sports than older people during the COVID-19 pandemic (McCarthy et al., 2021). Nevertheless, the literature provides mixed evidence on the issue at hand. Certain studies reported that, in contrast to young adults, older adults seem to significantly decrease their PA levels during the COVID-19 pandemic (Wunsch et al., 2022). These conflicting findings highlight the subtlety of the relationship between age group and SP, and the importance of additional work toward identifying the underlying mechanisms involved. At the individual behavioral level, Learning (Rank 1) and cultural lifestyle (Rank 7) represent foundational skills essential for enabling citizens to receive and act on vital information during the COVID-19 pandemic in order to engender greater resilience (Lopes and McKay, 2021). Adults who actively attend learning and cultural activities are likely to make informed health-related decisions (Lopes and McKay, 2020). In addition, since SP itself tends to be culturally integrated behavior (Macdonald et al., 2009), participants who engage in cultural and sport activities may belong to the same behavioral groups (Miles and Sullivan, 2012). Regular health examinations (Rank 6) were also found to be a significant factor. Individuals who have regular routine health examinations tend to be more attentive to their personal health (Yamaguchi et al., 2018), and those who are highly disease-aware are prone to have active lifestyles (Park et al., 2021). These findings lend support to the proposition that encouraging large-scale usage of health examinations can be an efficacious approach to promoting population-level health equity (Yoon et al., 2020). At the level of interpersonal relationships, Family/friend gatherings (Rank 4) represent the support from significant interpersonal networks that have been found to help adults maintain greater participation in sporting activities during the pandemic (Carter and Alexander, 2021). At the community environment level, a higher richness of facilities (Rank 9) is associated with adults spending more hours on sports activities (Liu et al., 2020). Thus, the versatility of sport environments appears significant in preventing the negative health effects of the pandemic (Virmasalo et al., 2023).

### 4.3 Policy implications and future preparedness

Common factor analysis, in combination with analysis of distinct factors, identifies that most major contributing factors are largely modifiable. For example, common factors such as recreational lifestyle, environmental dependency for PA, or individual initiative for SP are major areas for intervention. This exercise identifies that encouraging PA and SP in the context of a pandemic outbreak could be considered an appropriate concern for global health. It could provide critical direction to governments on designing programs aimed at preventing and controlling such behavioral patterns, thereby scaling up PA and SP.

First, to create the most optimal effect for health promotion policy, policymakers could concentrate efforts on those common key factors for PA and SP in the early stages post-outbreak. For instance, in our results indicating the importance of BMI, there is an unequivocal need to shift their agendas toward individualized management regarding weight, as well as addressing health behavior patterns, emphasizing those associated with changes in weight. Special attention should be paid to the impact of disrupted recreational rhythms under pandemic-related stress (Yang et al., 2021). Authorities must continually provide opportunities for adults to participate in recreational activities, leveraging their core role in processing complex realities, self, and society, while also regaining their sense of control (Ganter, 2025). Moreover, the suitability for exercise in the community environment, being the physical environment for health-centric activities, needs to be strategically planned. Community administrators should develop a preparatory checklist of preventive measures for surrounding exercise facilities to counter the risks of pandemic spread.

Second, in looking at PA promotion strategies, there is a need to emphasize efforts to mitigate environmental issues. As lockdowns implemented to reduce infections limit adults' access to fresh, healthy products (Di Renzo et al., 2020), we advocate for ensuring organized nutritional support (such as access to fresh food outlets) as a priority during isolations due to pandemics. On the other hand, policies should actively cultivate highly socially cohesive communities to help individuals cope with the stress of infection, improve views about the community, and hence create a virtuous cycle.

Third, to ensure adult SP behavior during a pandemic, there must be collaboration between governmental institutions and individuals. On one hand, although changes in lifestyle due to the COVID-19 outbreak (Musa et al., 2023) are considerable, there is a need to formulate policies to provide for an appropriate living environment, thereby ensuring that people practice preventive measures based on healthy living, diseases, and self-maintenance. The specific goal is to encourage individuals to enhance SP behavior through optimal overall health behaviors, thereby improving quality of life. On the other hand, participation in sports is also dependent on the level and nature of capital possessed by an individual (for example, cultural literacy, education level, income, and socio-economic status). Persons with low levels of socio-cultural capital could have limited awareness and utilization of health-improving aspects associated with SP. Economic constraints could also act as other hindrances. More strengthened sporting promotion policies are needed during pandemics. It is essential for policymakers to have a continuous concern for vulnerable groups to counter social inequalities in SP, which have worsened with the COVID-19 outbreak (Hoekman et al., 2024).

Finally, PA and SP outcomes are generated after being influenced by a multitude of other factors, which, in their complexity, could be captured in the context of a Socio-ecological Model. Such a model, which captures PA and SP in their entirety, in their biological, individual, behavioral, and environment-based contexts, would be crucial in comprehending PA and SP during any pandemic context. A thorough analysis of socio-ecological factors in specific critical periods will be vital for strengthening future public health initiatives and policymaking aimed at enhancing PA and SP levels (Zhu et al., 2024). Specifically, future efforts can leverage the power of artificial intelligence and interpretable machine learning (Rahayu and Ismail, 2023), as demonstrated in this study. These technologies, with their capacity to enable efficient data collection through intelligent analysis for predictions related to the outbreak of pandemics, as well as modeling health behavior, have great decision-making value for governments, health institutions, and individuals in their efforts to deal with pandemics.

### 4.4 Limitations and future research

This study has several limitations. It was first conducted exclusively from a nationally representative sample from China, and therefore limits the ability to generalize the findings broadly. Future research would be valuable in attempting to replicate the identified patterns in diverse global populations and cultural situations. Secondly, the cross-sectional nature of the CGSS2021 dataset did not allow for the drawing of strict causal links between the key influencing factors and PA or SP. Additionally, owing to data availability limitations, higher-level socio-ecological factors like public policy could not be integrated. Future research should utilize longitudinal tracking datasets to facilitate causal analysis as well as widen the range of influencing factors. Third, the level of PA and SP in the present study was assessed by a few items on the survey. Subsequent studies should take care to employ more multifaceted and extensive measures to support the validity of the assessment. Finally, a level of information redundancy was found amongst the socio-ecological factors used in the models. Future research should contemplate the use of feature selection methods before model building to facilitate the improvement in the explanatory ability of machine learning methods and focus on developing the best machine learning algorithms for PA and SP behaviors.

### 4.5 Contribution

This paper contributes to the literature in several ways. First, it proposes a socio-ecological approach to understanding adult PA and SP in the context of the COVID-19 pandemic, which, to the best of our knowledge, has not been done before. This model allows policymakers and practitioners to conduct a structured consideration of ways in which multilevel factors define PA behavior in situations of public health emergencies, thus better targeting more efficient distribution of intervention resources on those forces most likely to have a big impact on health. Second, the study draws on a nationally representative dataset. Differing from investigations utilizing small regional samples, results here provide wider generalizability and greater potential for informing policy at the national scale. Third, the study utilizes an interpretable machine learning method to discern the principal predictors of adult PA and SP. By not only enhancing the efficiency of extracting insight from large-scale datasets but also capturing nonlinear, complex associations between predictors and outcomes, this technique increases understanding of mechanisms underlying behavioral change and provides a robust analytical instrument for future investigations of social and health behaviors.

## 5 Conclusions

Drawing on the Socio-ecological Model and an interpretable machine learning approach, this study identified both common and distinct factors influencing PA and SP among adults during the COVID-19 pandemic. The main conclusions are as follows: (1) Common influences on both PA and SP during the COVID-19 pandemic were primarily rooted in the community context, particularly Suitability for Exercise, and were significantly shaped by Recreational lifestyle, with BMI emerging as a critical individual health indicator. These findings reinforce the notion that health-related behaviors are shaped by multi-level factors within the Socio-ecological Model. (2) PA during the COVID-19 pandemic was more strongly associated with external environmental and behavioral structuring factors, with distinct influences including Work status, Region, Family background, proximity of Fresh food outlets, Neighborhood help, Neighborhood care, as well as COVID-19 concern. In contrast, SP was more strongly driven by individual resources and behavioral initiative. Its distinct influences included Education level, Income level, engagement in Learning and Cultural lifestyle, regular Health examination, frequency of Family/Friend gatherings, and the Richness of facilities within the community. (3) In summary, during the COVID-19 pandemic, PA tends to be more passive and environmentally dependent, whereas SP is more indicative of personal initiative and the accumulation of socio-cultural and economic capital.

## Data Availability

Publicly available datasets were analyzed in this study. This data can be found here: https://www.cnsda.org/index.php?r=projects/view&id=65635422.
