# Supplementary material for: Multi-level determinants of physical activity and sports participation among adults during COVID-19 pandemic: an interpretable machine learning approach
Source: Front Psychol. 2026 Jan 7;16:1701201. doi: 10.3389/fpsyg.2025.1701201 (PMC12819603; doi:10.3389/fpsyg.2025.1701201)
Supplement: Supplementary file 1 [file Supplementary_file_1.docx]

Supplementary Material

# Supplementary Figures and Tables

## Supplementary Tables

**Supplementary Table 1.** Survey items and measurement scales of variables

| **Variables** | **Sample items** | **Sample scales** |
| --- | --- | --- |
| **Outcome Variables** |  |  |
| Sports participation | Over the past year, have you regularly participated in sports activities during your leisure time? | 0 = non-participation (including “several times a year or less” and “never”), 1 = participation (including “every day”, “several times a week”, and “several times a month”). |
| Physical activity | How long do you walk on a typical weekday? (including walking for work, daily living, commuting, and recreational purposes) And how many hours per week do you engage in physical activities that make you breathe faster than usual? (including jogging, cycling, exercising, carrying things, manual labor, and housework, but excluding general walking) | 0 = Not meeting standard (i.e., engaging in less than 150 minutes of physical activity per week), 1 = Meeting standard (i.e., engaging in 150 minutes or more of physical activity per week). |
| **Individual characteristics** |  |  |
| Sex | - | 1= Male, 2= Female |
| Age group | What is your date of birth? (Age was calculated by subtracting the year of birth from the year of the survey.) | 1 = Young adults (18–44 years), 2 = Middle-aged adults (45–59 years), 3 = Older adults (≥60 years). |
| Ethnic group | What is your ethnic group? | 1 = Han Chinese, 2 = Ethnic minorities |
| Religion | What is your religion? | 1=No religion, 2=Religion |
| Education level | What is your highest level of education completed? | 1 = Grade school or below (including no formal education, private school, literacy programs, grade school, and others), 2 = Junior high school, 3 = Senior high school (including vocational high school, general high school, secondary school, and technical school), 4 = Junior college or above (including junior college, undergraduate college, graduate degree, and higher) |
| Income level | What was your total personal gross income for the year 2020? | 1 = Below the 25th percentile of the sample, 2 = Between the 25th and 50th percentiles, 3 = Between the 50th and 75th percentiles, 4 = Above the 75th percentile |
| Political affiliation | What is your current political affiliation? | 1 = Non-Communist Party member, 2 = Communist Party member |
| BMI category | What is your current height? And what is your current weight? Body Mass Index (BMI) is calculated as weight (kg) divided by height squared (m²). | 1 = Underweight (BMI < 18.5), 2 = Normal weight (18.5 ≤ BMI < 24), 3 = Overweight (24 ≤ BMI < 28), 4 = Obese (BMI ≥ 28) |
| Health score | How would you rate your current state of health? In the past four weeks, how often have health problems interfered with your work or other daily activities? In the past four weeks, how often have you felt depressed or frustrated? Each item is rated on a 5-point Likert scale | These are treated as continuous variables (1-5). A composite health score is calculated by summing the responses to all three items and dividing by 3. Higher scores indicate better physical and psychological health status. |
| Household registration​ | What is your current household registration status? | 1 = Urban, 2 = Rural |
| Class identity | All things considered, where would you place yourself on the social ladder in the current society? (Please rate on a scale from 1 to 10, where 1 represents the lowest level and 10 represents the highest level of society.) | This is treated as a continuous variable (1-10). Higher scores indicate higher perceived social status. |
| Socioeconomic status | On balance, where would you place your own socio-economic status in the current society? | 1 = Lower class, 2 = Lower middle class, 3 = Middle class, 4 = Upper middle class, 5 = Upper class |
| Work status | What is your current work status? | 1 = Not working, 2 = Working |
| Region | Province | 1 = Eastern region (including Beijing, Tianjin, Hebei, Shanghai, Jiangsu, Zhejiang, Fujian, Shandong, Guangdong, Hainan, and Liaoning), 2 = Central region (including Shanxi, Anhui, Jiangxi, Henan, Hubei, Hunan, Jilin, and Heilongjiang), 3 = Western region (including Inner Mongolia, Guangxi, Chongqing, Sichuan, Guizhou, Yunnan, Xizang, Shaanxi, Gansu, Qinghai, Ningxia, and Xinjiang) |
| **Individual behaviors** |  |  |
| Traditional media use | In the past year, how frequently have you used the following types of media? (Newspapers, Magazines, Radio, Television) Each item is rated on a 5-point Likert scale (1 = Never, 5 = Very frequently). | These are treated as continuous variables(1-5). A composite media usage score is calculated by summing the responses to all four items and dividing by 4. Higher scores indicate more frequent media use. |
| New media use | In the past year, how frequently have you used the following types of media? (Internet, Mobile phones) Each item is rated on a 5-point Likert scale (1 = Never, 5 = Very frequently). | These are treated as continuous variables (1-5). A composite digital media usage score is calculated by summing the responses to both items and dividing by 2. Higher scores indicate more frequent media use. |
| Information source | Which of the following is your primary source of information? | 1 = Traditional media (including newspapers, magazines, radio, and television), 2 = New media (including the internet and mobile phones) |
| Recreational lifestyle | During the past year, how often have you engaged in the following leisure activities during your free time? (Watching TV or DVDs, Going out to the cinema, Shopping, Listening to music at home, Surfing the Internet) Each item is rated on a 5-point Likert scale (1 = Very frequently, 5 = Never). | These are treated as continuous variables (1-5). All responses are reverse-coded so that higher scores indicate a more active recreational lifestyle. A composite recreational lifestyle score is calculated by summing the reverse-coded responses and dividing by 5. |
| Cultural lifestyle | During the past year, how often have you engaged in the following leisure activities during your free time? (Reading books, newspapers, or magazines, attending cultural events (e.g., concerts, shows, exhibitions), doing crafts (e.g., embroidery, carpentry)) Each item is rated on a 5-point Likert scale (1 = Very frequently, 5 = Never). | These items are treated as continuous variables (1-5). All responses are reverse-coded so that higher scores indicate a more active cultural lifestyle. A composite cultural lifestyle score is calculated by summing the reverse-coded responses and dividing by 3. |
| Learning | During the past year, how often have you engaged in the following activities during your free time? (Learning) | 1 = Never, 2 = Rarely, 3 = Sometimes, 4 = Often, 5 = Frequently |
| Illness Status | Do you have any chronic illnesses or long-term health problems? | 1 = No, 2 = Yes |
| Smoking | Do you smoke? | 1 = Almost never or never smoked, 2 = Used to smoke, but do not smoke now, 3 = Currently smoke |
| Drinking | How often do you drink? | 1 = I don't drink, 2 = A few times a year or less, 3 = A few times a month, 4 = A few times a week, 5 = Every day |
| Health examination | Have you had any health examinations in the past three years? | 1 = No health examinations, 2 = Yes, but not on a regular basis, 3 = Yes, on a regular basis |
| **Interpersonal relationships** |  |  |
| Family/Friend gathering | During the past year, how often have you engaged in the following leisure activities during your free time? (Gathering with relatives who do not live with you, Gathering with friends) Each item is rated on a 5-point Likert scale (1 = Very frequently, 5 = Never). | These items are treated as continuous variables (1-5). All items are reverse-coded so that higher scores reflect greater engagement in family/friend gatherings. A composite score is calculated by summing the reverse-coded values and dividing by 2. |
| Social contact | During the past year, how often have you engaged in the following activities during your free time? (Social contact) | 1 = Never, 2 = Rarely, 3 = Sometimes, 4 = Often, 5 = Frequently |
| Neighbor socialising | How often do you engage in social and recreational activities with your neighbors (e.g., visiting each other’s homes, watching TV together, sharing meals, playing cards)? | 1 = Never, 2 = Once a year or less, 3 = A few times a year, 4 = About once a month, 5 = A few times a month, 6 = Once or twice a week, 7 = Almost every day |
| Family background | Thinking back to when you were 14 years old, where would you place your family on the social ladder? (Please rate on a scale from 1 to 10, where 1 represents the lowest level and 10 the highest level of society.) | This is treated as a continuous variable (1–10), with higher scores indicating higher perceived family background during adolescence. |
| Family economic status | Where would you place your household’s economic status relative to others in your local area? | 1 = Well below average, 2 = Below average, 3 = Average, 4 = Above average, 5 = Well above average |
| Household car | Does your household own a car? | 1 = No, 2 = Yes |
| Number of children | How many children do you have? | 1 = No children, 2 = One child, 3 = More than one child |
| Marital status | What is your current marital status? | 1 = No partner (including never married, separated but not divorced, divorced, widowed), 2 = Partnered (including cohabiting, first marriage with spouse, remarriage with spouse) |
| Family highest educational level | What is your father's highest level of education? What is your mother's highest level of education? (The higher of the two has been selected as the family’s highest educational level.) | 1 = Grade school or below (including no formal education, private school, literacy programs, grade school, and others), 2 = Junior high school, 3 = Senior high school (including vocational high school, general high school, secondary school, and technical school), 4 = Junior college or above (including junior college, undergraduate college, graduate degree, and higher) |
| **Community environment** |  |  |
| Social attitude | In general, do you agree that in this society, the vast majority of people can be trusted? In general, do you agree that in this society, if you are not careful, people will try to take advantage of you? In general, do you think today's society is fair? In general, do you think you are happy in your life? All items were measured on a 5-point Likert scale, treated as continuous variables ranging from 1 (strongly disagree) to 5 (strongly agree). | These items are treated as continuous variables (1-5). The second item was reverse-coded to ensure that higher values across all items consistently indicated more positive social attitudes. A composite social attitude score was computed by summing the values of the four items and dividing by four to yield an average score. |
| Social security | Are you currently enrolled in any of the following social security programs? (e.g., urban basic medical insurance, new rural cooperative medical insurance, publicly funded medical care, urban/rural basic pension insurance, commercial medical insurance, or commercial pension insurance). | 1 = not enrolled in any social security program, 2 = enrolled in at least one program. |
| COVID19 concern | How worried are you about catching COVID-19? | 1 = not at all worried, 2=not too worried, 3=somewhat worried, 4 = very worried |
| Neighborhood help | To what extent do you agree with the statement that the neighbors around me (within a one-kilometer radius, or about a 15-minute walk) care about each other? | 1 = Completely disagree, 2 = Disagree, 3 = Neither agree nor disagree, 4 = Agree, 5 = Completely agree |
| Neighborhood care | To what extent do you agree with the statement that the neighbors around me (within a one-kilometer radius, or about a 15-minute walk) are willing to help me when I am in need? | 1 = Completely disagree, 2 = Disagree, 3 = Neither agree nor disagree, 4 = Agree, 5 = Completely agree |
| Air pollution | How serious are the following problems where you live? (Air Pollution) | 1 = Not serious at all, 2 = Not too serious, 3 = Somewhat serious, 4 = Very serious |
| Water pollution | How serious are the following problems where you live? (Water pollution) | 1 = Not serious at all, 2 = Not too serious, 3 = Somewhat serious, 4 = Very serious |
| Noise pollution | How serious are the following problems where you live? (Noise pollution) | 1 = Not serious at all, 2 = Not too serious, 3 = Somewhat serious, 4 = Very serious |
| Suitability for Exercise | To what extent do you agree with the statement that the environment around me (within a one-kilometer radius, or about a 15-minute walk) is suitable for exercise, such as jogging or walking? | 1 = Completely disagree, 2 = Disagree, 3 = Neither agree nor disagree, 4 = Agree, 5 = Completely agree |
| Fresh food outlets | To what extent do you agree with the statement that there are plenty of fresh vegetables and fruit available around me (within a one-kilometer radius, or about a 15-minute walk)? | 1 = Completely disagree, 2 = Disagree, 3 = Neither agree nor disagree, 4 = Agree, 5 = Completely agree |
| Richness of facilities | To what extent do you agree with the statement that there are enough public facilities (e.g., community centers, libraries, parks) around me (within a one-kilometer radius, or about a 15-minute walk)? | 1 = Completely disagree, 2 = Disagree, 3 = Neither agree nor disagree, 4 = Agree, 5 = Completely agree |
| Safety | To what extent do you agree with the statement that I feel safe around where I live (within a one-kilometer radius, or about a 15-minute walk)? | 1 = Completely disagree, 2 = Disagree, 3 = Neither agree nor disagree, 4 = Agree, 5 = Completely agree |

**Supplementary Table 2.** Optimal parameters of eight mainstream machine learning algorithms

| **Algorithms** | **Coarse Search Range** | **Physical activity** | **Sports participation** |
| --- | --- | --- | --- |
|  |  | **Optimal parameters** | **Optimal parameters** |
| Logistic Regression | 'C': [0.01, 0.05, 0.1, 0.5, 1.0, 1.5], 'penalty': ['l1', 'l2'], 'solver': ['liblinear', 'lbfgs'],, 'max_iter': [100, 300, 500, 1000] | 'C': 0.05, 'max_iter': 100, 'penalty': 'l2', 'solver': 'lbfgs' | 'C': 1.2, 'max_iter': 100, 'penalty': 'l1', 'solver': 'liblinear' |
| Support Vector Machine | 'C': [0.01, 0.05, 0.1, 0.5, 1, 2], 'gamma': ['scale', 0.001, 0.01, 0.1, 1], 'kernel': ['linear', 'rbf'] | 'C': 0.01, 'gamma': 'scale', 'kernel': 'rbf' | 'C': 0.5, 'gamma': 0.01, 'kernel': 'rbf' |
| Decision Tree | 'criterion': ['gini', 'entropy'], 'max_depth': [3, 4, 5, 6, 8], 'min_samples_split': [2, 5, 10], 'min_samples_leaf': [1, 3, 5, 8], 'max_features': [None, 'sqrt', 'log2'] | 'criterion': 'entropy', 'max_depth': 4, 'max_features': None, 'min_samples_leaf': 5, 'min_samples_split': 5 | 'criterion': 'gini', 'max_depth': 4, 'max_features': None, 'min_samples_leaf': 1, 'min_samples_split': 2 |
| Random Forest | 'n_estimators': [100, 200, 300, 400, 500], 'criterion': ['gini', 'entropy'], 'max_depth': [5, 10, 15, 20], 'max_features': ['sqrt', 'log2'], 'min_samples_split': [2, 4, 6], 'min_samples_leaf': [1, 2, 4, 6], 'bootstrap': [True, False] | 'bootstrap': True, 'criterion': 'entropy', 'max_depth': 10, 'max_features': 'sqrt', 'min_samples_leaf': 4, 'min_samples_split': 2, 'n_estimators': 275 | 'bootstrap': True, 'criterion': 'gini', 'max_depth': 15, 'max_features': 'sqrt', 'min_samples_leaf': 4, 'min_samples_split': 2, 'n_estimators': 435 |
| Gradient Boosting | 'n_estimators': [50, 100, 200, 300], 'learning_rate': [0.01, 0.05, 0.1], 'max_depth': [2, 3, 4], 'min_samples_split': [2, 4, 6], 'min_samples_leaf': [1, 3, 6], 'subsample': [0.6, 0.8, 1.0] | 'loss': 'log_loss', 'learning_rate': 0.01, 'max_depth': 2, 'min_samples_leaf': 6, 'min_samples_split': 2, 'n_estimators': 221, 'criterion': 'friedman_mse', 'subsample': 0.6 | 'loss': 'log_loss', 'learning_rate': 0.05, 'max_depth': 3, 'min_samples_leaf': 1, 'min_samples_split': 2, 'n_estimators': 110, 'criterion': 'friedman_mse', 'subsample': 0.6 |
| Adaptive Boosting | 'n_estimators': [10, 50, 100, 150, 200], 'learning_rate': [0.01, 0.03, 0.05, 0.1] | 'n_estimators':16, 'learning_rate':0.03, 'algorithm':'SAMME.R', 'base_estimator':'deprecated' | 'learning_rate': 0.01, 'n_estimators': 170, 'algorithm':'SAMME.R', 'base_estimator':'deprecated' |
| eXtreme Gradient Boosting | 'n_estimators': [50, 100, 200, 300], 'learning_rate': [0.01, 0.05, 0.1], 'max_depth': [3, 4, 5, 6], 'gamma': [0, 0.3, 0.7, 1.0], 'min_child_weight': [1, 3, 5], 'subsample': [0.6, 0.8, 1.0], 'colsample_bytree': [0.8, 1.0] | 'colsample_bytree': 1.0, 'gamma': 0.7, 'learning_rate': 0.01, 'max_depth': 3, 'min_child_weight': 5, 'n_estimators': 206, 'subsample': 1.0 | 'colsample_bytree': 1.0, 'gamma': 1, 'learning_rate': 0.05, 'max_depth': 6, 'min_child_weight': 1, 'n_estimators': 50, 'subsample': 1.0 |
| Light Gradient-Boosting Machine | 'n_estimators': [30, 50, 100, 200], 'learning_rate': [0.05, 0.1, 0.25], 'max_depth': [4, 6, 8, 10, 12], 'num_leaves': [2, 10, 20, 30] 'subsample': [0.6, 0.8, 1.0], 'colsample_bytree': [0.8, 1.0] | 'boosting_type': 'gbdt', 'colsample_bytree': 1.0, 'learning_rate': 0.25, 'max_depth': 6, 'n_estimators': 50, 'num_leaves': 2, 'subsample': 0.6 | 'boosting_type': 'gbdt', 'colsample_bytree': 1.0, 'learning_rate': 0.11, 'max_depth': 11, 'n_estimators': 34, 'num_leaves': 23, 'subsample': 0.6 |
